# Supplementary figures and images for: A non-enveloped arbovirus released in lysosome-derived extracellular vesicles induces super-infection exclusion
Source: PLoS Pathog. 2020 Oct 19;16(10):e1009015. doi: 10.1371/journal.ppat.1009015 (PMC7595637; doi:10.1371/journal.ppat.1009015)

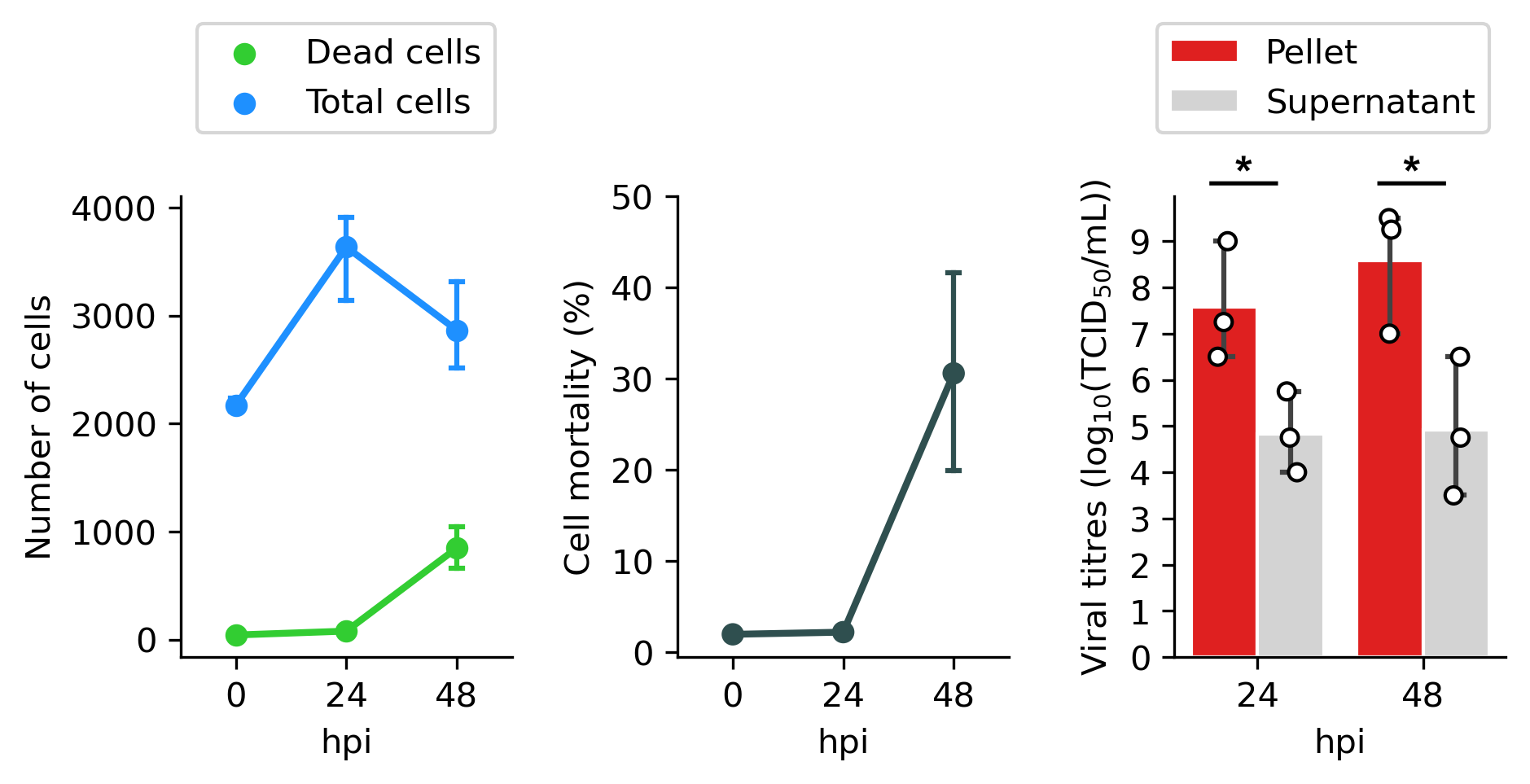

Supplement: S1 Fig — The number of dead cells versus the total number of cells was measured (left panel) in our infected cell cultures, to calculate the percentage of cell mortality (middle panel) at the time of infection (0 hpi), at 24 hpi and at 48 hpi. In parallel, we measured BTV viral titres in the pellet or the supernatant after a 10,000xg centrifugation in viral suspension harvested from infected cells at 24 hpi or 48 hpi (right panel). The 24 hpi time point represented the optimal balance between cell mortality and viral titres, and was used for all the subsequent experiments. Data are presented as mean ± SD. Each point indicates the value of independent replicates (N = 3; unpaired t-test, *p<0.05). (TIF) [file ppat.1009015.s001.tif]

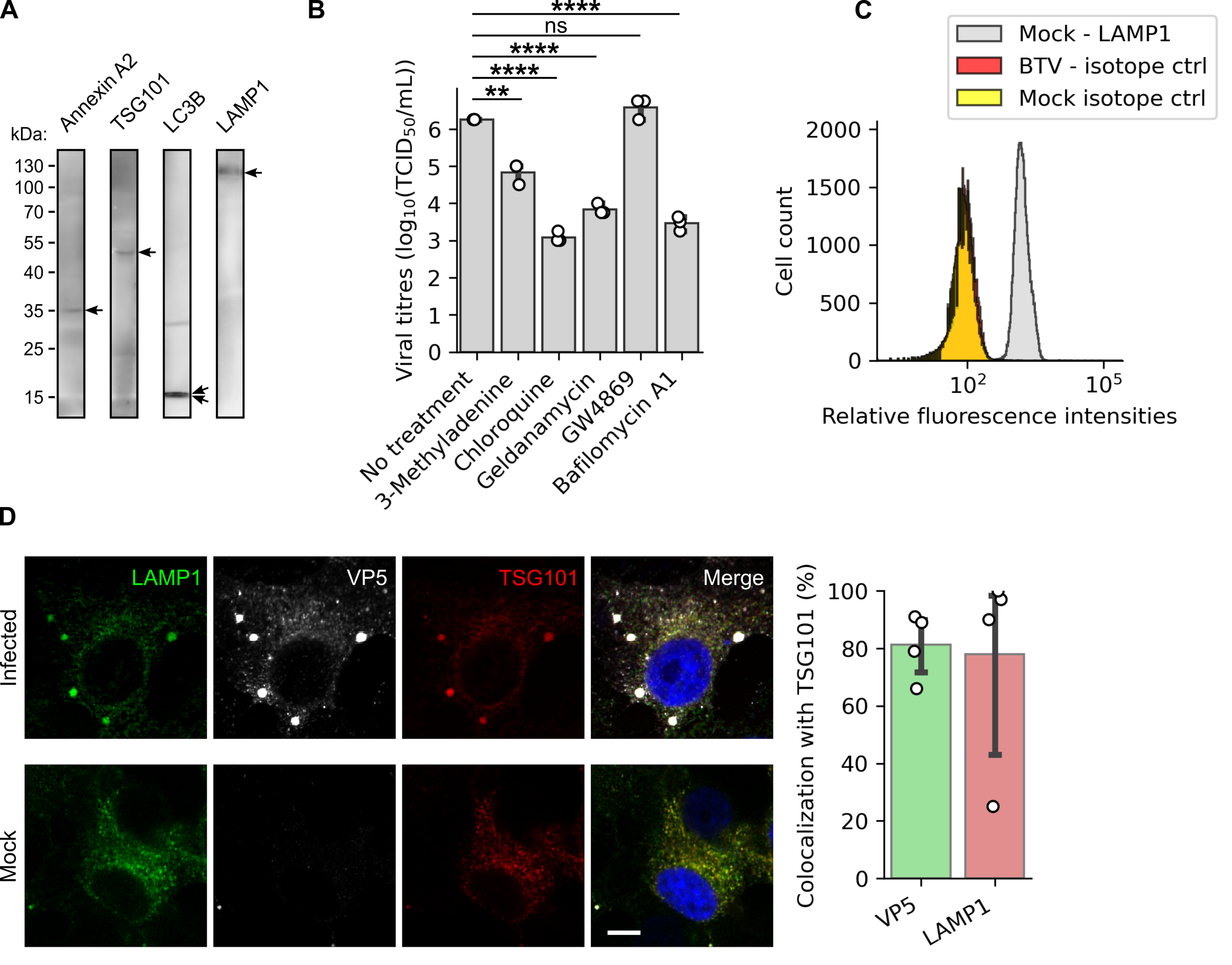

Supplement: S2 Fig — (A) Western blot detection of cellular proteins Annexin A2, TSG101, LC3B and LAMP1 in EVs released from non-infected sheep cells. (B) Intracellular viral titres measured at 24 hpi in sheep infected cells in presence or absence of 3-MA, CQ, geldanamycin, GW4869 on bafilomycin A1. Data are presented as mean ± SD. Each point indicates the value of independent replicates (N = 3, unpaired t-test, ns p>0.05, **p<0.01, ****p<0.0001). (C) Determination of the background fluorescence in mock and BTV infected sheep cells using an isotope control antibody. For reference, the level of fluorescence is also represented in mock cells labelled with a LAMP1 antibody. (D) Microscopy images of mock or BTVWT infected sheep cells labelled with anti LAMP1, VP5 and TSG101 antibodies. Scale bar: 5 μm. Right panel show the percentage of VP5 and LAMP1 co-localising with TSG101 in infected cells. (TIF) [file ppat.1009015.s002.tif]

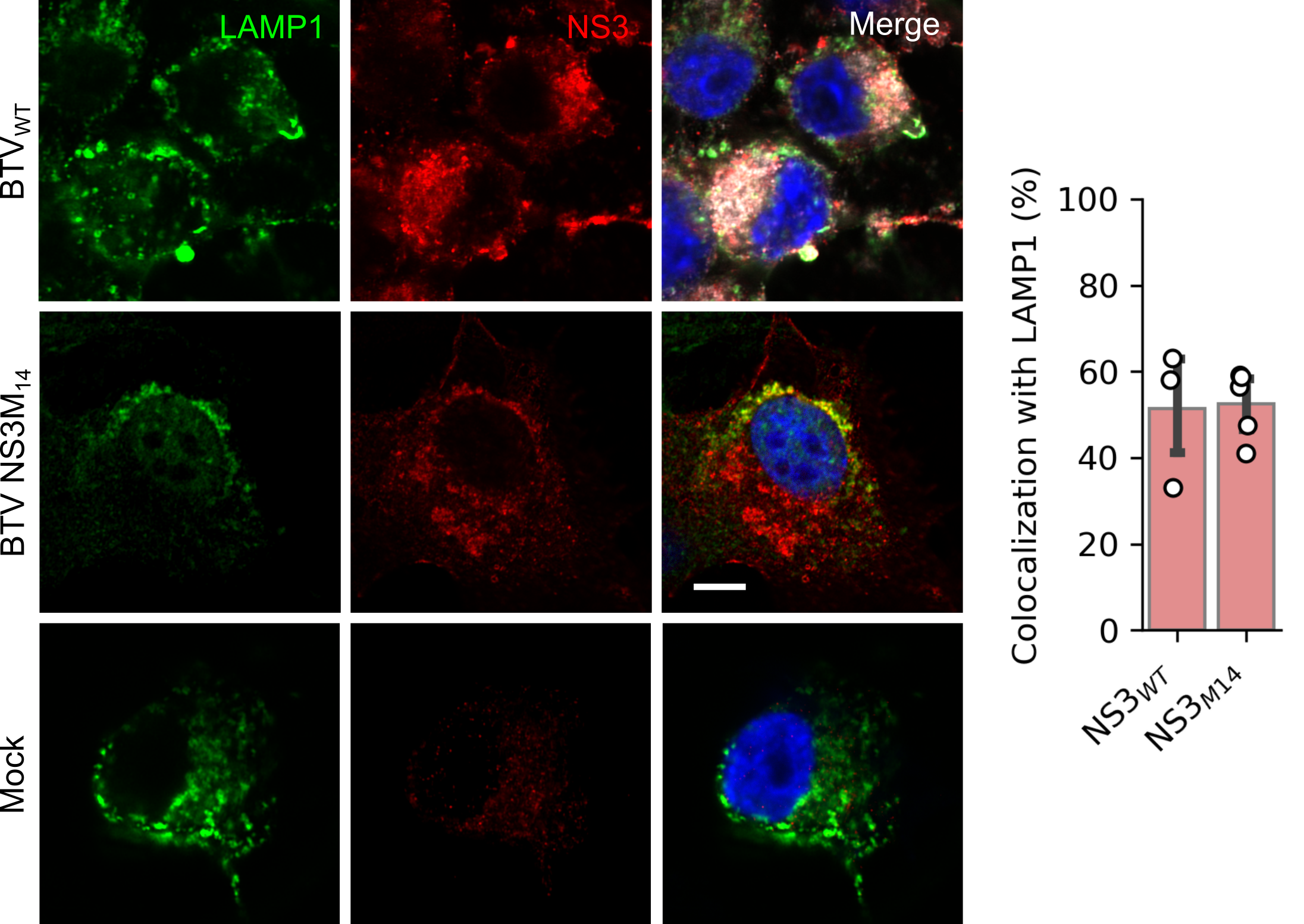

Supplement: S3 Fig — Microscopy images of mock, BTVWT, or BTV NS3M14 infected sheep cells labelled with anti LAMP1 and NS3 antibodies. Scale bar: 5 μm. Right panel show the percentage of NS3WT and NS3M14 co-localising with LAMP1 in infected cells. (TIF) [file ppat.1009015.s003.tif]
