# Supplementary material for: A non-enveloped arbovirus released in lysosome-derived extracellular vesicles induces super-infection exclusion
Source: PLoS Pathog. 2020 Oct 19;16(10):e1009015. doi: 10.1371/journal.ppat.1009015 (PMC7595637; doi:10.1371/journal.ppat.1009015)
Supplement: S1 Table — (PDF) [file ppat.1009015.s004.pdf]

Hpi:	6h	9h	12h	15h	18h	21h
EVs infected cells (N_{VIBs})	429	423	416	1020	982	912
Free virus infected cells (N_{VIBs})	149	106	187	314	367	509

S1 Table. Total number of lysosomes analysed per time point in sheep cells infected with EVs or free virus particles.
